# Effect of Dupilumab on Allergen-Specific IgE Levels and Clinical Tolerance in Patients with Food Allergy: A Systematic Review

**DOI:** 10.3390/ijms27177670

**Published:** 2026-08-27

**Authors:** Magdalena Grześk-Kaczyńska, Szymon Kaczyński, Bernadetta Kosztulska, Magdalena Rydzyńska, Tomasz Rosada, Natalia Ukleja-Sokołowska

**Affiliations:** 1 Department and Clinic of Allergy, Clinical Immunology and Internal Diseases, Collegium Medicum in Bydgoszcz, Nicolaus Copernicus University in Torun, 87-100 Torun, Poland; 2 Department of Obstetrics, Gynecology and Gynecological Oncology, Faculty of Medicine, Collegium Medicum in Bydgoszcz, Nicolaus Copernicus University in Torun, 87-100 Torun, Poland

**Keywords:** dupilumab, food hypersensitivity, desensitization, immunologic

## Abstract

Food allergy is a growing global health concern associated with significant dietary restrictions, impaired quality of life, and risk of severe allergic reactions. Dupilumab, a monoclonal antibody targeting the interleukin-4 receptor alpha (IL-4Rα), inhibits IL-4 and IL-13 signaling and has demonstrated efficacy across multiple type 2 inflammatory diseases. This systematic review aimed to evaluate the effects of dupilumab on food allergy outcomes in pediatric and adult patients. Across studies, dupilumab treatment was consistently associated with reductions in total immunoglobulin E (IgE) and food-specific IgE levels, indicating modulation of allergic sensitization and type 2 inflammation. However, these immunologic improvements were not consistently accompanied by clinically meaningful desensitization or acquisition of food tolerance. In a phase II trial of peanut allergy, dupilumab monotherapy reduced immunologic markers but did not improve oral food challenge outcomes. When combined with oral immunotherapy, dupilumab modestly enhanced desensitization rates but did not substantially reduce treatment-related allergic reactions. Current evidence suggests that dupilumab favorably affects immunologic markers associated with food allergy, particularly total and allergen-specific IgE levels. Nevertheless, there is insufficient evidence to conclude that dupilumab alone induces sustained clinical tolerance to food allergens. Larger randomized controlled trials incorporating standardized oral food challenges and long-term follow-up are required to clarify the clinical role of dupilumab in food allergy management.

## 1. Introduction

Dupilumab is a fully human monoclonal antibody of the IgG4 subclass that blocks signaling of interleukin-4 (IL-4) and interleukin-13 (IL-13) by binding to the shared IL-4 receptor α (IL-4Rα) subunit. IL-4 and IL-13 are central mediators of type 2 inflammation and contribute to IgE production, eosinophilic inflammation, and disruption of epithelial barrier function [1,2]. By inhibiting IL-4/IL-13 signaling, dupilumab suppresses type 2 inflammatory responses and may therefore target several mechanisms implicated in the pathophysiology of food allergy.

Food allergy is characterized by allergen-specific type 2 immune responses, including production of food-specific IgE, activation of mast cells and basophils, and eosinophilic inflammation. IL-4 and IL-13 promote IgE class switching and contribute to impaired epithelial barrier function, potentially facilitating allergen sensitization and persistence of allergic inflammation. These mechanisms provide a biological rationale for investigating dupilumab as a potential therapeutic option in food allergy [2,3].

Dupilumab has demonstrated clinical efficacy across several type 2 inflammatory diseases, including atopic dermatitis, asthma, chronic rhinosinusitis with nasal polyps, and eosinophilic esophagitis [4,5,6,7], supporting the broader therapeutic relevance of IL-4/IL-13 blockade [8]. Its favorable safety profile, with injection-site reactions, conjunctivitis, and transient eosinophilia among the most commonly reported adverse events, further supports its investigation in food allergy [5].

Food allergy affects an estimated 6–10% of the global population and represents an increasing public health burden worldwide. Recent epidemiological data indicate that food allergy affects approximately 6–8% of children and up to 10% of adults in developed countries, although prevalence varies depending on diagnostic criteria and geographic region [9]. Clinical manifestations range from mild, localized reactions such as oral allergy syndrome (OAS) to systemic, potentially life-threatening anaphylaxis. The unpredictable nature of reactions and the constant risk of accidental exposure significantly impair quality of life, contributing to anxiety, social limitations, and increased caregiver burden [10].

Current management of food allergies is primarily based on strict elimination diets and patient education. While effective in preventing reactions, elimination strategies are difficult to maintain and may negatively impact nutritional status, particularly in children. In recent years, oral immunotherapy (OIT) has emerged as a disease-modifying treatment approach, particularly for peanut, milk, and egg allergy. OIT can increase the threshold of reactivity and reduce the risk of severe reactions after accidental exposure, although treatment is associated with frequent adverse events and requires prolonged maintenance therapy [11].

Recently, biological therapies have been introduced into the management of food allergies. Omalizumab, an anti-IgE monoclonal antibody, was approved by the U.S. Food and Drug Administration in 2024 for use in patients with IgE-mediated food allergy to reduce the risk of allergic reactions following accidental exposure. It is indicated particularly in individuals with multiple food allergies or those at high risk of severe reactions and has been shown to increase tolerance thresholds and improve the safety of immunotherapy.

A major concern in food allergy management is the use of multi-elimination diets, which may lead to nutritional deficiencies, impaired growth in children, and reduced dietary diversity, further exacerbating psychosocial burden [10]. These challenges highlight the need for targeted therapies that address the underlying immunological mechanisms.

Dupilumab may represent a promising therapeutic option in food allergy due to its ability to suppress type 2 inflammation. By inhibiting key cytokine signaling involved in IgE class switching, eosinophilic inflammation, and epithelial barrier dysfunction, dupilumab could potentially reduce allergen sensitization and clinical reactivity. Although its role in food allergy is still under investigation, emerging evidence suggests that modulation of the IL-4/IL-13 axis can substantially reduce total and food-specific IgE levels, providing a biological rationale for investigating its potential clinical effects in this population [12].

The aim of this systematic review was to evaluate the effects of dupilumab on food allergy outcomes, including immunologic markers and clinical reactivity, in pediatric and adult patients with atopic diseases.

## 2. Materials and Methods

**Figure 1:**
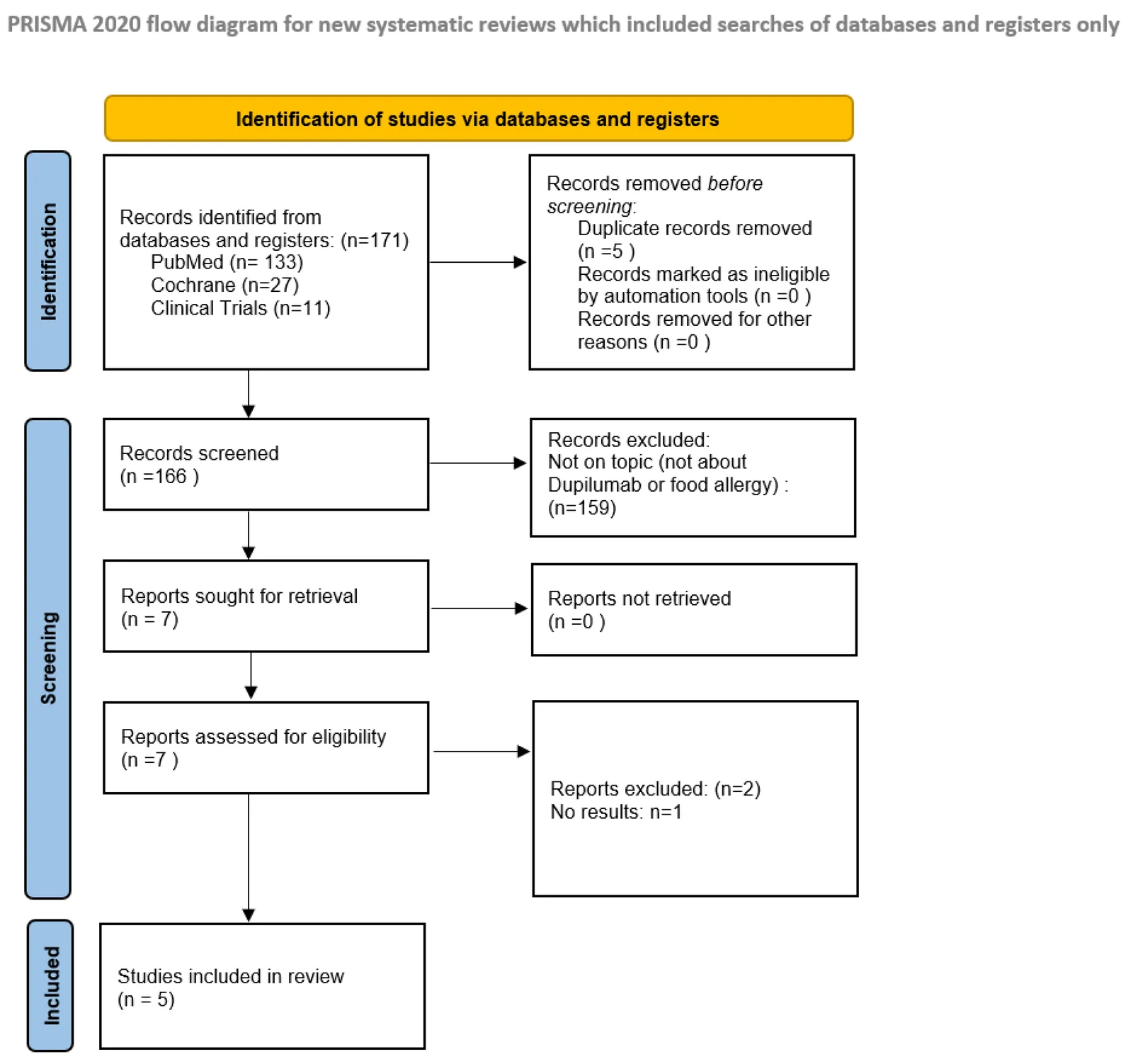
Identification of studies via databases and registers. The PRISMA 2020 statement.

This systematic review was conducted in accordance with PRISMA 2020 guidelines. A systematic search of PubMed, Cochrane CENTRAL, and ClinicalTrials.gov was performed on 25 April 2026. The search strategy was based on the combination of the terms “food allergy” AND “dupilumab”; no additional filters were applied. A total of 171 records were identified, and 166 remained after duplicate removal. Following title and abstract screening, 160 records were excluded in compliance with the Preferred Reporting Items for Systematic Reviews and Meta-Analyses (PRISMA) 2020 guidelines, presented in the PRISMA 2020 flow diagram [Figure 1]. Five full-text articles were assessed for eligibility, all of which met inclusion criteria and were included in the qualitative synthesis. Two reviewers independently screened titles, abstracts, and full texts. Disagreements were resolved by discussion or consultation with a third reviewer. The protocol was prospectively registered in the Open Science Framework (DOI: 10.17605/OSF.IO/X4D5Y).

Studies were included if they met the following criteria:

- Were published within the last 5 years;
- Included pediatric and/or adult populations;
- Evaluated treatment with dupilumab in the context of food allergy;
- Reported changes in food allergy–related outcomes before and after treatment, including serum allergen-specific IgE (sIgE);
- Were randomized controlled trials or observational studies;
- Were published in English.

The methodological quality and risk of bias of the included studies were assessed using the Cochrane risk-of-bias tools. The ROBINS-I tool (Risk Of Bias In Non-randomized Studies of Interventions) was used to evaluate non-randomized studies, while randomized controlled trials were assessed using the Cochrane Risk of Bias 2 (RoB 2) tool. Reporting bias was not formally assessed because fewer than ten studies were included.

The findings of the included studies were synthesized narratively and are presented in summary tables. Studies were compared by study design, patient characteristics, intervention protocols, efficacy outcomes, and immunological markers. Owing to the clinical and methodological heterogeneity among the included studies, quantitative synthesis (meta-analysis) was not performed.

## 3. Results

A total of five studies met the inclusion criteria and were included in the qualitative synthesis, analyzing (*n* = 288) patients, among whom the majority were minors (*n* = 243) [13,14,15,16,17] [Table 1]. The included studies comprised randomized controlled trials and observational studies and involved both pediatric and adult populations with food allergy and/or atopic dermatitis. Across observational studies conducted in patients with atopic dermatitis and concomitant food allergy, dupilumab treatment was associated with reductions in immunologic markers of allergic sensitization, including decreases in total IgE and sIgE levels. These findings were reported by Emerson et al., Sernicola et al., and van der Rijst et al. and supported by post hoc analyses of dupilumab-treated pediatric atopic dermatitis cohorts [13,14,17]. However, reductions in IgE were not consistently associated with improved clinical food tolerance [13,15]. Risk-of-bias assessment showed that the overall methodological quality of the included studies was moderate. The observational studies were assessed using ROBINS-I and were judged to have a moderate risk of bias owing to their non-randomized design, small sample sizes, absence of control groups, and potential selection bias. Across all studies, heterogeneity in food allergy diagnosis and the limited use of oral food challenges reduced the certainty of the available evidence [Table 2].

Despite this heterogeneity, methodological strengths included the use of multicenter designs, randomized placebo-controlled methodology, and standardized DBPCFC endpoints in the studies directly evaluating peanut allergy, as well as longitudinal assessment of immunologic outcomes in observational cohorts [15,16,17]. However, these strengths were limited by small sample sizes, lack of control groups in most observational studies, and inconsistent assessment of clinical outcomes.

A retrospective single-center observational study by Emerson et al. [13] evaluated the effect of dupilumab treatment on food allergy outcomes in children and adolescents with atopic dermatitis and concomitant food allergy (*n* = 60; age range: 6 months–18 years). Patients received dupilumab 200 mg every 2 weeks or 300 mg every 4 weeks, depending on the weight. Food allergy diagnosis was based on clinical history, sIgE, skin prick testing, and oral food challenges when clinically indicated. Dupilumab treatment was associated with a decrease in sIgE levels over time (−0.6% per month; 95% CI −0.8 to −0.4; *p* < 0.001), while no significant changes were observed in skin prick test reactivity. Oral food challenge outcomes remained comparable to previously published real-world cohorts, with an approximately 76% pass rate and no clear evidence of improved clinical tolerance attributable to dupilumab. Overall, the study suggests that dupilumab may reduce immunologic sensitization markers but does not appear to substantially alter the clinical course of food allergy. The main sources of bias included selection bias, confounding, the absence of a control group, the retrospective study design, and the fact that oral food challenges were performed in only a subset of patients. According to the ROBINS-I tool, the overall risk of bias was assessed as moderate.

A prospective observational pilot study by Sernicola et al. [14] evaluated the effect of dupilumab treatment in polysensitized adult patients with atopic dermatitis and concomitant food allergy (*n* = 20; age ≥ 18 years). Food allergy diagnosis was based on clinical history supported by sIgE testing (ImmunoCAP). Patients received dupilumab at a dose of 600 mg followed by 300 mg every 2 weeks for at least 16 weeks. Treatment was associated with reductions in total IgE levels, sIgE, and sensitization to aeroallergens. No food-related adverse reactions were reported despite an unrestricted diet during treatment. The main sources of bias included the small sample size, lack of randomization, absence of a control group, unrestricted diet during the study period, and the lack of systematic oral food challenges (OFCs). According to the ROBINS-I tool, the overall risk of bias was assessed as moderate.

Sindher et al. [15] conducted a multicenter, open-label, phase II clinical study to evaluate the efficacy and safety of dupilumab in children with peanut allergy. The primary endpoint was the proportion of participants who achieved tolerance to a predefined dose of peanut protein during an oral food challenge (OFC) after completion of treatment.

The study demonstrated that dupilumab monotherapy did not result in clinically meaningful desensitization in the majority of participants, despite reductions in peanut-specific IgE and changes in immune biomarkers. The safety profile was consistent with the established safety profile of dupilumab, with most adverse events being mild to moderate. The authors concluded that dupilumab alone may have limited efficacy as a monotherapy for peanut allergy and suggested further evaluation in combination approaches, such as with oral immunotherapy. The study was considered to be at high risk of bias because of the lack of randomization and blinding. The observational studies were assessed using ROBINS-I and were judged to have a moderate risk of bias owing to their non-randomized design, small sample sizes, absence of control groups, and potential selection bias.

Chinthrajah et al. (2025) [16] conducted a multicenter, randomized, placebo-controlled study to evaluate whether dupilumab added to peanut OIT improves treatment outcomes in children with peanut allergy. Participants received either dupilumab plus OIT or placebo plus OIT, and efficacy was assessed using an OFC.

The study found that adding dupilumab to OIT slightly improved desensitization outcomes compared with OIT alone, resulting in a higher proportion of children achieving the target peanut dose. However, the overall clinical benefit was limited. The combination treatment was generally well tolerated, and no new safety concerns were reported.

van der Rijst et al. (2024) [17] conducted an observational study to evaluate the effect of dupilumab on sIgE levels in pediatric patients with atopic dermatitis. Children receiving dupilumab were followed over time, and changes in serum levels of sIgE were assessed for common food allergens.

The study demonstrated that dupilumab treatment was associated with a significant reduction in sIgE levels during follow-up. Decreases were observed across multiple food allergens, suggesting that inhibition of type 2 inflammation may influence allergic sensitization. However, the study did not evaluate clinical food tolerance or confirm resolution of food allergy through oral food challenges. The authors concluded that dupilumab may reduce immunological markers of food allergy, although further studies are needed to determine whether these changes translate into clinical improvement. The main sources of bias included the observational study design, the absence of a control group, and the lack of systematic assessment of clinical tolerance using oral food challenges. According to the ROBINS-I tool, the overall risk of bias was assessed as moderate.

## 4. Discussion

The present systematic review adds to the existing literature by integrating the currently available clinical and observational evidence on dupilumab in patients with food allergy and/or atopic dermatitis across pediatric and adult populations. Our synthesis indicates that the most consistent effect of dupilumab is the reduction in total and food-specific IgE, whereas evidence for clinically meaningful desensitization or food tolerance remains limited and inconsistent. Importantly, the available data suggest that this discrepancy is particularly relevant for dupilumab monotherapy, while combination with oral immunotherapy may provide greater clinical benefit. By highlighting the dissociation between immunologic and clinical outcomes, as well as the importance of treatment duration and the unresolved question of treatment timing, this review identifies key gaps that should guide future clinical studies.

The present review suggests that dupilumab consistently reduces immunologic markers associated with allergic sensitization in patients with atopic dermatitis and concomitant food allergy [13,15]. Across the included studies, dupilumab treatment was associated with reductions in both total IgE and sIgE levels, supporting the role of IL-4 and IL-13 blockade in modulating type 2 inflammatory responses. These findings were observed in both pediatric and adult populations and were consistent across observational studies and clinical trials, making the reduction in sIgE one of the most reproducible immunologic effects of dupilumab reported to date. However, reductions in sIgE were not consistently accompanied by corresponding changes in skin prick test reactivity or other markers of sensitization across all studies [13].

In contrast to the consistent biomarker improvements, the clinical outcomes were considerably less convincing. Reductions in total and sIgE levels were not consistently accompanied by clinically meaningful desensitization, increased reaction thresholds during oral food challenges, or acquisition of food tolerance [13,15]. The lack of a consistent relationship between declining IgE levels and clinical food tolerance may be explained by the fact that circulating sIgE represents only one component of the allergic effector response. Clinical reactivity depends not only on the concentration of circulating allergen-specific IgE but also on the amount of IgE bound to high-affinity receptor for the Fc region of immunoglobulin E (FcεRI) on mast cells and basophils, the functional responsiveness of these effector cells, and local tissue inflammatory mechanisms. Moreover, IL-4 and IL-13 may contribute to mast-cell activation and FcεRI expression through mechanisms that are not entirely dependent on circulating IgE levels [18]. Thus, a substantial reduction in serum sIgE may occur without a corresponding increase in the threshold for clinical reactions. This may explain why marked reductions in total and peanut-specific IgE after dupilumab monotherapy in the study by Sindher et al. did not translate into clinically meaningful desensitization [15]. Regarding clinical reactivity, current evidence does not demonstrate a consistent increase in reaction thresholds or reduction in reaction severity with dupilumab monotherapy. The available clinical data are limited, while a modest improvement in desensitization has been observed when dupilumab is combined with oral immunotherapy [15,16].

Furthermore, sIgE levels do not always correlate with true clinically relevant allergies, and declining sIgE concentrations alone do not indicate resolution of food allergies. This is particularly important in studies in which oral food challenges, the current gold standard for confirming food allergy and assessing the development of tolerance, were not systematically performed. Similarly, food-specific IgE measurements and skin prick tests primarily identify sensitization and, when used in isolation, have limited specificity for predicting clinical reactivity [19].

From the authors’ perspective, the limited clinical efficacy of dupilumab monotherapy suggests that suppression of type 2 inflammation alone may be insufficient to induce clinically meaningful desensitization in established food allergy. Despite substantial reductions in total and peanut-specific IgE, only 2 of 24 children (8.3%) achieved the predefined DBPCFC endpoint after 24 weeks [15]. The somewhat greater desensitization observed with dupilumab combined with peanut oral immunotherapy suggests that dupilumab may be more useful as an adjunctive immunomodulatory treatment than as monotherapy for inducing food tolerance [16].

Moreover, most of the included studies were observational, involved relatively small patient populations, and showed considerable heterogeneity regarding age groups, study design, diagnostic criteria, and outcome assessment. In many studies, food allergy diagnosis was based primarily on clinical history and sensitization testing without systematic confirmation by oral food challenge, which remains the gold standard for diagnosis and assessment of tolerance. Most studies were conducted in patients with atopic dermatitis, making it difficult to determine whether observed effects were directly related to food allergy modulation or secondary to improvement of skin barrier dysfunction and systemic type 2 inflammation [20].

Two things should be taken into consideration: the optimal duration of dupilumab treatment and the preferred time of treatment induction in the prevention of further sensitization.

The duration of dupilumab treatment for food allergy remains uncertain. Available pediatric data suggest that immunologic responses may evolve with treatment duration. In a study by Sindher et al., 24 weeks of dupilumab treatment resulted in substantial reductions in total IgE and peanut-specific IgE; however, only 2 of 24 participants (8.3%) achieved the predefined clinical endpoint of passing the DBPCFC with ≥444 mg of peanut protein, indicating that biomarker improvement did not translate into clinically meaningful desensitization within 24 weeks [15]. Conversely, a retrospective cohort of children and adolescents with food allergy receiving dupilumab for atopic dermatitis demonstrated a 0.6% decrease in food-specific IgE for each additional month of treatment, suggesting a time-dependent immunologic effect [13]. However, the relationship between longer treatment duration and clinical food tolerance could not be established.

In adult patients, the available evidence suggests that immunologic changes related to food allergen sensitization may become evident within the first 16 weeks of dupilumab treatment. In a prospective observational pilot study of 20 adults with moderate-to-severe atopic dermatitis and documented severe food allergy, 16 weeks of dupilumab treatment was associated with significant reductions in total IgE (median 654 to 446 kU/L) and food-specific IgE (median 8.02 to 1.48 kUA/L), accompanied by a reduction in molecular food allergen-specific IgE [14]. However, oral food challenges were not performed, and therefore these findings cannot establish the timing of clinical improvement in food allergy, desensitization, or acquisition of food tolerance. Although no food-related clinical symptoms were reported during the observation period, this finding should be interpreted cautiously because food exposure was not standardized or challenge-confirmed.

Regarding the preferred timing of dupilumab initiation, there are currently no clinical data or established evidence indicating when dupilumab should be initiated in patients with atopic dermatitis (AD) to achieve a preventive effect on food allergy. Nevertheless, the timing of treatment may be particularly relevant in young patients with severe AD, given the close relationship between skin-barrier dysfunction, type 2 inflammation, and the development of food sensitization. The dual-allergen-exposure hypothesis proposes that exposure to food allergens through an impaired and inflamed skin barrier may promote epicutaneous sensitization, whereas early oral exposure promotes the development of immune tolerance [21,22]. Early-onset and more severe AD are associated with a higher risk of food sensitization and food allergy, suggesting that the period of active skin inflammation may represent an important window during which sensitization can occur [22,23]. Therefore, early and effective control of AD, including restoration of skin-barrier integrity and suppression of type 2 inflammation, could theoretically reduce the opportunity for further epicutaneous sensitization to food allergens. In this context, dupilumab may potentially have an additional effect beyond controlling cutaneous inflammation, as inhibition of IL-4/IL-13 signaling may influence the immunological mechanisms involved in IgE production. In pediatric patients with AD, dupilumab treatment has been associated with substantial reductions in food-specific IgE, and the authors proposed that treatment at a younger age might potentially influence the development of food allergy through earlier improvement of epidermal barrier dysfunction and reduction in type 2 inflammation [17]. However, these observations remain hypothesis-generating, and there is currently no evidence demonstrating that earlier initiation of dupilumab prevents de novo food sensitization or the subsequent development of clinical food allergy. Thus, the optimal timing of dupilumab initiation from a food allergy prevention perspective remains unknown and should be addressed in prospective studies, particularly in young children with severe AD who are at high risk of developing food sensitization.

Overall, current evidence suggests that dupilumab can reduce biomarkers of allergic sensitization, particularly total and sIgE levels. However, the available evidence does not consistently demonstrate corresponding improvements in clinically relevant outcomes, including reaction thresholds, desensitization, or long-term food tolerance. More large, well-designed studies are needed to determine whether the observed immunological changes translate into meaningful clinical benefit in patients with food allergy.

In recent years, several biologic therapies have been introduced or are being investigated for the treatment of food allergy. These therapies target different pathways involved in allergic inflammation and may offer new treatment options for patients with food allergy, either alone or in combination with oral immunotherapy.

Food allergy is associated with a significant burden for patients, families, and healthcare systems. Dietary restrictions, fear of accidental reactions, reduced quality of life, and healthcare costs contribute to its social and socioeconomic impact. Therefore, there is a clear need to develop safe and effective treatments that can improve quality of life and reduce the burden of disease. Future research should focus on identifying therapies that not only modify immunological markers but also provide meaningful clinical benefits for patients.

Currently, biologic therapy for food allergy is rapidly evolving. Omalizumab became the first biologic approved by the U.S. Food and Drug Administration (FDA) in February 2024 for reducing allergic reactions following accidental exposure in patients aged ≥1 year with IgE-mediated food allergy. In the phase III OUtMATCH (Omalizumab for the Treatment of Multiple Food Allergies) trial, 67% of participants receiving omalizumab achieved the predefined threshold of at least 600 mg of peanut protein compared with 7% receiving placebo, with similar benefits observed for several other food allergens [24,25]. Omalizumab was generally well tolerated, with safety outcomes comparable to placebo apart from more injection-site reactions. Compared with dupilumab, omalizumab currently has stronger clinical evidence for increasing reaction thresholds, whereas dupilumab has demonstrated more consistent reductions in total and food-specific IgE but limited efficacy as monotherapy [15]. Dupilumab was also well tolerated in the available food allergy trials, with a safety profile consistent with its established use in atopic dermatitis [15,16]. However, neither agent has yet been shown to induce durable food tolerance after treatment discontinuation, and no direct head-to-head comparison between omalizumab and dupilumab is available.

Beyond omalizumab, several biologic agents are currently under clinical investigation, including ligelizumab, tezepelumab, and therapies targeting the IL-33/ST2 pathway [26,27,28]. These agents are being evaluated both as monotherapy and as adjuncts to oral immunotherapy, with the aim of improving desensitization, reducing treatment-related adverse events, and ultimately achieving sustained unresponsiveness.

Etokimab, an anti-IL-33 monoclonal antibody, showed promising results in a phase IIa randomized, placebo-controlled trial in adults with peanut allergy. A single dose increased the tolerated peanut dose during oral food challenge, reduced peanut-specific IgE levels, and was well tolerated. However, due to the small sample size and short follow-up, further studies are needed before its role in food allergy can be established [28].

Tezepelumab is currently being evaluated in an ongoing phase II randomized trial as an adjunct to peanut oral immunotherapy [29].

Ligelizumab, a next-generation anti-IgE monoclonal antibody, has been evaluated in a phase III clinical trial in patients with IgE-mediated peanut allergy, including long-term extension studies. At the time of this review, however, the available evidence was limited, and definitive conclusions regarding its efficacy in food allergy could not be drawn [30].

Although preliminary results are promising, particularly regarding immunological modulation, robust evidence demonstrating durable clinical tolerance and long-term disease modification remains limited. Therefore, at present, omalizumab is the only approved biologic for IgE-mediated food allergy, whereas all other biologics should still be considered investigational for this indication.

This systematic review has several limitations. First, only a small number of studies met the inclusion criteria, reflecting the limited evidence currently available on dupilumab in food allergy. Second, considerable heterogeneity existed among the included studies with respect to study design, patient populations, underlying atopic diseases, diagnostic criteria for food allergy, treatment duration, and reported outcomes, which precluded quantitative meta-analysis. In addition, most studies included patients with atopic dermatitis and concomitant food allergy rather than individuals with isolated food allergy, limiting the generalizability of the findings. Finally, oral food challenges—the gold standard for confirming food allergy and assessing the development of clinical tolerance—were not systematically performed in most studies, making it difficult to determine whether reductions in immunologic markers translated into meaningful clinical benefit.

## 5. Conclusions

Current evidence suggests that dupilumab may influence immune responses in patients with food allergy and atopic dermatitis, with several studies reporting reductions in total IgE and food-specific IgE levels. However, the clinical significance of these immunologic changes remains uncertain, and current evidence is insufficient to determine whether dupilumab monotherapy can induce clinically meaningful or sustained food tolerance. Larger randomized controlled studies with oral food challenges and longer follow-up are needed to determine whether these immunologic changes result in lasting clinical improvement.

## Figures and Tables

**Table 1:** The summary table of included articles.

Outcomes	Intervention	Diagnosis of Food Allergy	Population	Type of the Study	Name of the Study	#
Clinical: OFC outcomes: ~76% pass rate, consistent with real-world populations Biomarkers: sIgE decreased by 0.6% for each additional month of treatment, whereas SPT ^6^ did not differ. Follow-up: variable; median 17 months (sIgE) and 14.5 months (SPT)	Dupilumab > 6 months as treatment for AD, 200 mg q2w ^1^ 300 mg q4w ^1^	SPT ^6^, sIgE ^4^, oral food challenges (both performed in clinic and at home through home introduction or accidental ingestion)	*n* = 60 children Age: 6 months–18 years old	Retrospective single-center observational study	Food allergy clinical course in children and adolescents treated with dupilumab for atopic dermatitis [13].	1
Clinical: no reported food reactions (unrestricted diet) No OFC performed Biomarkers: ↓ Total IgE ↓ sIgE ^4^ (food + aeroallergens) ↓ sensitization (SPT ^6^) Follow-up: 16 weeks	Dupilumab dose: 600 mg loading dose followed by 300 mg q2w ^1^ for 16 weeks or longer	Clinical history + positive allergy testing: SPT ^6^ (positive if wheal ≥ 3 mm), sIgE ≥ 0.35 kUA/L, and OFC when needed. CRD ^5^ used in some patients	*n* = 20 adults >18 years old, Polysensitized	Prospective observational pilot study	Dupilumab as Therapeutic Option in Polysensitized Atopic Dermatitis Patients Suffering from Food Allergy [14].	2
Clinical: no improvement in DBPCFC ^3^; no increase in tolerated dose; no desensitization observed Biomarkers: ↓ total IgE; ↓ peanut-sIgE ^4^; Follow-up: 36 weeks	Dupilumab 300 mg or 200 mg for 24 weeks, q2w ^1^	Physician-confirmed peanut allergy with clinical reaction history, peanut-specific IgE positivity, and objective reactivity during DBPCFC ^3^ at screening	*n* = 24 children Age: 6–17 years Confirmed peanut allergy DBPCFC ^3^	Phase 2, multicenter, open-label study	Efficacy and Safety of Dupilumab in Children With Peanut Allergy: A Multicenter, Open-Label, Phase II Study [15].	3
Clinical: ↑ desensitization (vs. placebo + OIT) no reduction in OIT-related allergic reactions similar safety profile between groups Biomarkers: ↓ total IgE; ↓ peanut-specific IgE; ↓ peanut-specific IgG4/IgE ratio; ↓ basophil activation Follow-up: 12 weeks post-treatment	Dupilumab + peanut OIT vs. placebo + OIT; SC q2w ^1^ (weight-based dosing); OIT up-dosing (12 → 300 mg) followed by maintenance (300 mg/day); DBPCFC	Clinical history, peanut-specific IgE sensitization, and screening DBPCFC ^3^	*n* = 148 enrolled; 123 analyzed; children and adolescents with peanut allergy Age: 6–17 years old	Phase 2 randomized, placebo-controlled trial (adjunct to OIT)	Dupilumab as an Adjunct to Oral Immunotherapy in Pediatric Patients With Peanut Allergy [16].	4
Clinical: No data Biomarkers: ↓ food-specific IgE ↓ total IgE Follow-up: 1 year	No data	sIgE ^4^ levels and clinical history	*n* = 36 children Age: 4–17 years AD ^2^ + Food allergy	Observational cohort study	Dupilumab induces a significant decrease of food specific immunoglobulin E levels in pediatric atopic dermatitis patients [17]	5

^1^ q2w—once every 2 weeks; q4w—once every 4 weeks; ^2^ AD—atopic dermatitis; ^3^ DBPCFC—double-blind placebo-controlled food challenge; ^4^ sIgE—serum food-specific immunoglobulin E; ^5^ CRD—component-resolved diagnostics; ^6^ SPT—skin prick test; ↓—decrease; ↑—increase.

**Table 2:** The risk-of-bias assessment.

#	Name of the Study	Tool	D1 Randomization	D2 Deviations from Intended Intervention	D3 Missing Outcome Data	D4 Outcome Measurement	D5 Selection of Reported Results	Overall
1	Food allergy clinical course in children and adolescents treated with dupilumab for atopic dermatitis [13]	ROBINS-I	-	-	-	-	-	Moderate
2	Dupilumab as Therapeutic Option in Polysensitized Atopic Dermatitis Patients Suffering from Food Allergy [14]	ROBINS-I	-	-	-	-	-	Moderate
3	Efficacy and Safety of Dupilumab in Children With Peanut Allergy: A Multicenter, Open-Label, Phase II Study [15]	RoB 2	Some concerns	High	Low	Low	Some concerns	High risk
4	Dupilumab as an Adjunct to Oral Immunotherapy in Pediatric Patients With Peanut Allergy [16]	RoB 2	Low	Low	Some concerns	Low	Low	Some concerns
5	Dupilumab induces a significant decrease of food specific immunoglobulin E levels in pediatric atopic dermatitis patients [17]	ROBINS-I	-	-	-	-	-	Moderate

## Data Availability

No new data were created or analyzed in this study. Data sharing is not applicable to this article.

## Abbreviations

The following abbreviations are used in this manuscript:

AD	Atopic dermatitis
CRD	Component-resolved diagnostic
DBPCFC	Double-blind placebo-controlled food challenge
FcεRI	High-affinity receptor for the Fc region of immunoglobulin E
IgE	Immunoglobulin E
IL-4	Interleukin-4
IL-13	Interleukin-13
IL-4Rα	Interleukin-4 receptor α
OAS	Oral allergy syndrome
OFC	Oral food challenge
OIT	Oral immunotherapy
OUtMATCH	Omalizumab for the Treatment of Multiple Food Allergies
PRISMA	Preferred Reporting Items for Systematic Reviews and Meta-Analyses
ROBINS-I	Risk of Bias in Non-randomized Studies of Interventions
sIgE	Serum food-specific immunoglobulin E
SPT	Skin prick test
